# How to identify and recruit nurses to a survey 14 and 24 years after graduation in a context of scarce data: lessons learnt from the 2012 nurses at work pilot study on nurses’ career paths

**DOI:** 10.1186/s12913-015-0787-2

**Published:** 2015-03-26

**Authors:** Véronique Addor, André Jeannin, Diane Morin, Philippe Lehmann, Floriane Roulet Jeanneret, René Schwendimann

**Affiliations:** Haute Ecole de Santé Genève, HES-SO, Avenue de Champel 47, 1206 Genève, Switzerland; Unit of Prevention Programmes’ Evaluation, Institute of Social and Preventive Medicine (IUMSP), Centre Hospitalier Universitaire Vaudois and University of Lausanne, Lausanne, Switzerland; Institut Universitaire de Formation et de Recherche en Soins, University of Lausanne, Lausanne, Switzerland; Haute Ecole de Santé Vaud (HESAV), HES-SO, Lausanne, Switzerland; Haute Ecole de Santé Genève, Avenue de Champel 47, 1206 Genève, Switzerland; Institute of Nursing Science, University of Basel, Basel, Switzerland

**Keywords:** Nursing workforce, Career, Pilot study, Switzerland, Cohort, Shortage, Health systems

## Abstract

**Background:**

Nursing workforce data are scarce in Switzerland, with no active national registry of nurses. The worldwide nursing shortage is also affecting Switzerland, so that evidence-based results of the nurses at work project on career paths and retention are needed as part of the health care system stewardship; nurses at work is a retrospective cohort study of nurses who graduated in Swiss nursing schools in the last 30 years. Results of the pilot study are presented here (process and feasibility).

The objectives are (1) to determine the size and structure of the potential target population by approaching two test-cohorts of nursing graduates (1988 and 1998); (2) to test methods of identifying and reaching them 14 and 24 years after graduation; (3) to compute participation rates, and identify recruitment and participation biases.

**Methods:**

Graduates’ names were retrieved from 26 Swiss nursing schools: 488 nurses from the 1988 cohort and 597 from 1998 were invited to complete a web-based questionnaire. Initial updated addresses (n = 278, seed sample) were found using the Swiss Nursing Association member file. In addition, a snowball method was applied for recruitment, where directly-contacted respondents provided additional names of graduate mates or sent them the invitation. The study was further advertized through the main employers, study partners, and a press release.

**Results:**

Participation rate was 26.5% (n = 287), higher for the older cohort of 1988 (29.7%, n = 145) than for 1998 (15.6%, n = 93). Additional nurses (n = 363) not belonging to the test cohorts also answered. All schools were represented among respondents. Only 18 respondents (6%) worked outside nursing or not at all. Among respondents, 94% would ‘probably’ or ‘maybe’ agree to participate in the main study.

**Conclusion:**

The pilot study demonstrated that targeted nurses could be identified and approached. There is an overwhelming interest in the project from them and from policymakers. Recommendations to increase nurses’ participation rate for nurses at work include: (1) to open nurses at work recruitment to all nurses in Switzerland, while recreating cohorts post-hoc for relevant analysis; (2) to define a comprehensive communication strategy with special attention to graduate nurses who are harder to reach.

## Background

The current worldwide nursing imbalances and shortages rank high in the agenda of stakeholders such as the WHO [1,2], OECD [3], EU [4], ICN [5], Ministries of Health, and health institutions [6]. Shortages have been experienced recurrently since the 1930s due to the increase in the demand of care (demographic and epidemiologic transitions, technological advances) [7,8]. Shortage is now projected to increase in all ageing countries, mainly due to the increasing prevalence of chronic diseases at all ages, the decrease in informal care provided by families, shorter hospital stays, substitution of inpatient with outpatient care, tasks-shifting from physicians to nurses, the ageing of the nursing workforce itself [9,10] and insufficient number of graduates mainly due to demographic shifts.

It is well documented that insufficient nursing staffing significantly worsens the quality of health care, and increases patients’ morbidity and mortality [11-15]. Nursing-sensitive outcomes such as nosocomial infections, pressure wounds, falls, failure-to-rescue for surgical complications, or pain management are significantly linked with nursing shortage [16,17]. The quantitative and qualitative adequacy of nursing resources must therefore be considered as a part of health systems’ response to the demand for high quality health care, as it is a central objective of any health system [17,18].

However, the factors that attract or discourage nurses are culturally-sensitive, and most studies have been conducted in English-speaking countries and/or outside Europe, often with major limitations in terms of length of follow-up (one to three years), design (cross-sectional), type of settings (mainly acute care hospitals), measurement constructs (ex: ‘intention to leave’ among nurses currently working in nursing/health care institutions as a proxy for actual quitting [19,20]), and the exclusion of nurses working outside nursing or outside the workforce [21-25], except in the U.S. [26-28] and Australia [29,30]. To our knowledge, only one research team (The RN Work Project) is currently working prospectively on this topic with three successive nursing graduate cohorts recruited throughout the U.S. since 2004; this on-going prospective cohort study of nurses from all work settings – similarly to nurses at work - shows that 18% of new RN graduates actually leave their first nursing employer within one year, 26% within two year (with 92% taking another nursing job with a different employer), and 60% within eight years; this Project deals with a number of the above-mentioned biases [31-33].

While increasing recruitment into nursing is obviously crucial, it cannot counterbalance attrition from the workforce, in particular the loss of experienced nurses and/or those educated at the tertiary level to who then qualify for better employment plans outside nursing [34]. Despite high implementation costs for governments and institutions, retention strategies in specific institutions as well as in the health sector in general have not been systematically evaluated [35-37]. The practice of recruiting nurses abroad is widespread in Switzerland, with annual nurse migration net growth e.g. from Germany, France and Italy [38], but it has logistical, political, professional and ethical limitations [39-41].

Data on nursing in Switzerland are perhaps even more limited than in most industrialized countries, in part due to the fact that education and certification in nursing is the responsibility of the cantons, not the Federal government. Even the number of nurses currently working in the Swiss health system is unknown, precluding precise forecasting and effective health system stewardship [10,42,43]. The Swiss Health Observatory estimated that compared to 2006, between 25,000 and 48,000 additional health professionals (an increase of 13-25%) will be needed by the year 2020 just in hospitals, home care and nursing homes, but the proportion of nurses from this estimate cannot be determined because of the multiplicity of terms used for ‘nurse’ and a lack of clarity in the classification used to describe diplomas in the health sector [10]. Fresh graduates from Swiss nursing schools will cover only half to one third of the immediate needs [43].

The nurses at work project focuses on the retention aspect of the nursing shortage, as this has been identified in the literature as the main single determinant of the stock of nurses in the labor market [2,3,10], and on graduates ‘produced’ by the Swiss education system. The specific nurses at work literature review and corresponding choice of measurement instruments will be published elsewhere. Nurses at work is designed to better understand career paths of nurses who graduated in Swiss nursing schools in the last 30 years by measuring duration of employment and identifying reasons to stay in or leave (a) the profession, (b) a sector of care, or (c) an institution. This article reports on the recruitment and feasibility aspects of the nurses at work pilot study, which main objectives were (1) to establish the most effective methods of identifying and reaching the target population, (2) to determine the size and structure of the target population, (3) to describe the characteristics of respondents, including potential participation rates and possible biases.

## Methods

The size of the nurses at work main study’s total target population over the last three decades was unknown at the start of the pilot study, i.e. nurses from seven cohorts of graduation (1978, 1983, 1988, 1993, 1998, 2003, and 2008) who obtained their initial diploma from nursing schools in Switzerland (whatever their nationality). Besides the lack of a national registry of nurses, other major problems in reconstituting cohort sizes (i.e. denominators for computing participation rates) were that there is no systematic tracing of the opening, termination or merging of nursing schools over the years, and that nursing curricula and diploma names had changed four times over the period of interest.

The 30-year time frame was chosen as to cover the potential range of professionally active nurses including at least three generations of nurses, and allowing for studying changes in medium-term (5-year) paths across all cohorts and longer-term paths in earlier cohorts. In addition, the 5-year intervals should limit possible historical biases due to the merging and changes in curricula in certain years, not always occurring simultaneously in all cantons and nursing schools. There are three main languages in Switzerland, corresponding to cultural regions: German (about 70% of the population), French (about 25%) and Italian (about 5%). For the pilot study, we were able to identify and survey all schools in the French- and Italian-speaking regions, but we had to select a convenience sample from the German-speaking part including two large groups of nursing schools of Bern and Basel due to limited resources.

Data collection efforts for denominators (obtaining lists of graduates) were concentrated on two test cohorts of 1988 and 1998. However, all information that we could come across about the seven cohorts were collected for future use in the nurses at work main study. Cohort-specific participation rates were computed using respondents to the survey as numerators.

The Ethics Committee of Vaud (whose authority covers the whole country through a ‘working community of ethical research’) approved the protocol of the pilot study on November 25, 2011.

### Data sources

#### Primary data sources

‘Primary lists’ (i.e. names of graduates and contact details at the time of graduation for all seven cohorts of interest) were elaborated with the 26 nursing schools that we identified as having awarded diplomas to at least one of the seven cohorts between 1978 and 2008: 22 schools in the French-speaking region, 2 in the German-speaking region (resulting from the merging of several schools), and 2 in the Italian-speaking region. These primary lists were compared with the centralized files of nursing diplomas of the Swiss Red Cross (SRC, paper files only) for the validation of denominators, since all nursing schools had to register diplomas with the SRC at that time. Additional potential sources were investigated: the Swiss Federal Office of Statistics, the Swiss Federal Office of Public Health, and Cantonal Public Health Departments (which give the authorization to practice nursing), but no relevant data were available.

#### Secondary data sources

Various ‘secondary’ data sources were tested to update contact details for the two test cohorts: the ‘white pages’ internet directories, the Swiss Nursing Association (ASI/SBK) membership files, individual nursing schools’ societies of alumni (most of which do not exist anymore), and main employers such as hospitals, home care and school/community health services. The most valid secondary source was the ASI/SBK members’ file, which improved contact information for 25.6% of nurses from the primary lists who belonged to the two test cohorts. These nurses became our seed sample (n = 278) for the pilot survey. However, an important drawback is that the vast majority of ASI/SBK members are nurses who are currently working in nursing.

#### Snowball procedure

Nurses identified in primary and secondary listings were used as informants in a snowball procedure. They were asked if they still had contacts with graduate mates and if they were willing either to provide us directly with potential respondents’ contact details or to communicate with their graduate mates to invite them to participate in the nurses at work pilot study.

### Reaching and recruiting the target population

#### Direct contact

All addresses were checked or completed using the internet directory (i.e. white/yellow pages). Out of the 278 invitations sent in the three official languages to the nurses from the ASI/SBK seed sample, 135 were for the 1988 cohort and 143 for the 1998 cohort. Only 15 envelopes (5.4%) came back as unknown. Overall, 115 contacts in the ASI/SBK file comprised an email address. Since both types of invitations (i.e. email and postal) might have been sent to the same person, a warning was posted on all study material asking to participate only once. Two email reminders were sent to all seed and referred contacts. The snowball method was optimized by sending daily – postal and/or email- invitations to all new contacts we received.

#### Other types of contacts

Human Resources and Nursing Directorates of 88 health institutions (see employers above) were contacted. Their participation was requested for posting an advertisement on their intranet/extranet, and eventually sending an invitation to participate to all targeted nurses working in their institution, based on graduation year. The nurses at work study also asked 35 study partners, including present nursing schools’ Directors, to advertize the pilot survey.

A secured nurses at work website (http://www.nurses-at-work.com ) and email address were developed. Specific information regarding the objectives of the study, informed consent and confidentiality were made available in each of the three languages. Hyperlinks to - and information on - the questionnaire were also included. Therefore, nurses could access the questionnaire without having been invited formally and even if their names were not in primary or secondary listings. In addition, a Facebook page for nurses at work was created, including a short summary of the project, pictures of the study team, links to and from the website, as well as links to recent articles related to nursing shortage. A press release was sent to 24 newspapers, 10 radio stations and 5 TV channels in the French-speaking region; to 16 newspapers, 14 radio stations and 11 TV channels in the German-speaking region; as well as to 22 newspapers, 3 radio stations and 2 TV channels in the Italian speaking-region. Unfortunately, we could not obtain free and timely support from our communication services, so that no personal contact could be made with any journalist.

### The nurses at work questionnaire

#### Content, development and pretest

The main objectives of the pilot study’s questionnaire were to test recruitment and participation, as well as acceptability and interest of the target population for the topic. A secondary objective was to pretest understanding and acceptability of the survey’s specific questions on nurses’ career paths. Study-tailored questions were developed by the nurses at work team. Socio-economic items and the list of health sectors of employment were partially adapted from national surveys conducted by the Swiss Federal Office of Statistics.

A pre-test for face validity, understanding, acceptability and administration time was performed, first on a paper version, then on a revised electronic version, with two convenience samples of five nurses each, eight of them teaching at HEdS and two in clinical settings, males and females of various ages. As a result, changes were made in clarifying what we intend by ‘nurse’, this term encompassing not only clinical positions, but also nursing teachers and managers in nursing or in the health sector. The online survey was developed on LimeSurvey© (www.limesurvey.org).

The questionnaire (available on request) finally comprised a total of 28 often multi-item questions: 7 pertained to nursing education, 12 to career paths (based on our conceptual framework, results will be published separately), 2 to identification with nursing, four to nurses at work main study (intention to participate, network, and source of invitation to the pilot study), and 3 to their socio-demographic situation and contact details. Space for comments was provided at the end of the questionnaire. As part of the snowball procedure, respondents were invited to provide contact information about graduate mates by completing a form inserted into the questionnaire.

#### Data collection and analysis

The online survey ran for 9 weeks from May 31 to August 7, 2012; there was no paper version. In order to simplify access to the survey, we did not send a personal identifier to potential respondents. The warning to participate only once was efficient inasmuch as there were no duplicate questionnaires. We purposely did not force answers to any questions such as graduation year or canton. The questionnaire had to be completed within one online session. Data analysis included descriptive statistics and analysis of variance as appropriate to answer the study questions. All statistical analyses were performed using IBM SPSS Statistics (version 20.0; IBM Inc., Armonk, NY, USA).

## Results

### Size and structure of the target population

Individual nursing schools provided 1085 unduplicated names for the two test cohorts: 488 nurses for the 1988 graduation year and 597 nurses for 1998 (the two test cohorts.) To ascertain the quality of these denominators in the absence of a gold standard - such as a national registry of nurses -, we compared the raw number of graduates for each school/cohort combination with the corresponding figures retrieved from the SRC archives, the discrepancies – 8.9% nurses missing in the SRC files – may be due to clerical error and were randomly distributed (data not shown).

Considering the whole range of graduation files from all nursing schools and all cohorts, we observed a wide diversity in available formats: out of a total of 138 files of annual graduation cohorts containing 3,187 unduplicated names of graduates (including the 1085 members of the two test cohorts), 69% were in Excel format, 18% were in png format (mainly for the 1978 cohort), 8% were in pdf format, and 5% were in Word format.

### Identifying and reaching graduates from the two test cohorts

Out of the 1085 nurses who belonged to the pilot study target population, 287 participated in the survey (26.5%): 180 from the 1988 cohort (36.9%) and 107 from the 1998 cohort (17.9%). However, only 238 provided their school name, canton and graduation year (Table 1). Participation rates by region - therefore conservative – were higher in French-speaking cantons. The highest participation rate of 41.9% (n = 18) was found in canton Fribourg for 1988 (data not shown), whereas we have not explanation for the square absence of Italian-speaking respondents from the 1998 cohort (see Table 2). We did not have enough resources to track individual contact details, with multiple address changes including in the new names provided by invited respondents. Therefore, it is not possible to disentangle those, among the 287 respondents, who belonged to the original invitees from the ASI/SBK seed file from those who came into the study through the snowball procedure, as they could be invited by multiple sources.

**Table 1:** **Participation to nurses at work pilot survey by region**

	**1988**			**1998**			**Total**		
**Region**	**Lists**	**Respondents**	**Participation**	**Lists**	**Respondents**	**Participation**	**Lists**	**Respondents**	**Participation**
	**N**	**n**	**%**	**N**	**n**	**%**	**N**	**n**	**%**
French	352	111	31.5	230	56	24.3	582	167	28.7
German	102	24	23.5	352	37	10.5	454	61	13.4
Italian	34	10	29.4	15	0	0.0	49	10	20.4
Total	488	145	29.7	597	93	15.6	1,085	238	21.9

Region is based on the canton where the nursing school was located; only respondents who provided their nursing school name are included in this table. Lists = number of graduates from graduation lists.

**Table 2:** **Current professional situation, by graduation cohort and region (n = 287)**

		**Active in nursing**		**Active outside nursing**		**Non active**		**Total**	
		**n**	**%**	**n**	**%**	**n**	**%**	**n**	**%**
French	1988	121	93.1	5	3.8	4	3.1	130	100.0
	1998	60	96.8	2	3.2	0	0	62	100.0
	**Total**	**181**	**94.3**	**7**	**3.6**	**4**	**2.1**	**192**	**100.0**
German	1988	32	97.0	1	3.0	0	0	33	100.0
	1998	37	90.2	3	7.3	1	2.4	41	100.0
	**Total**	**69**	**93.2**	**4**	**5.4**	**1**	**1.4**	**74**	**100.0**
Italian	1988	9	81.8	1	9.1	1	9.1	11	100.0
	1998	0	0	0	0	0	0	0	0
	**Total**	**9**	**81.8**	**1**	**9.1**	**1**	**9.1**	**11**	**100.0**
Total	1988	162	93.1	7	4.0	5	2.9	174	100.0
	1998	97	94.2	5	4.9	1	1.0	103	100.0
	**Total**	**259**	**93.5**	**12**	**4.3**	**6**	**2.2**	**277**	**100.0**

Base: respondents from the two test cohorts.

About 80% of respondents completed the questionnaire in 16 minutes or less. Due to the absence of a personal identifier, access to the survey questionnaire was unrestricted. As many as 930 entries were registered on LimeSurvey©, of which 280 were excluded because they remained less than one minute on the questionnaire and did not answer any question (we hypothesized that they were either employers wanting to check the seriousness of the study before advertising it, or nurses from other cohorts). In addition to the 287 respondents from the two test cohorts, as many as 363 persons ‘forced themselves’ through the first selection questions because they heard from the study one way or the other; they were subsequently classified as belonging to ‘other cohorts’. Some of them indicated in the free comments section that they had completed their nursing education abroad but were working in Switzerland, others that they were interested in the survey, or were asked by their employer to participate anyway. We decided to include this latter group in Table 3.

**Table 3:** **Non-exclusive sources of the invitation to participate, by year of graduation (n = 650)**

**Sources**	**1988**		**1998**		**Other**		**Total**	
	**n**	**%**	**n**	**%**	**n**	**%**	**n**	**%**
Email	95	22.3	59	13.8	118	27.7	272	63.8
Employer	38	8.9	19	4.5	48	11.3	105	24.6
Letter from nurses at work	65	15.3	23	5.4	7	1.6	95	22.3
Colleague	4	0.9	6	1.4	11	2.6	21	4.9
Graduate mates	8	1.9	2	0.5	0	.0	10	2.3
Ad or media article	3	0.7	4	0.9	5	1.2	12	2.8
Alumni association	1	0.2	1	0.2	1	0.2	3	0.7
Facebook	0	0	0	0	1	0.2	1	0.2
Other mean	2	0.5	3	0.7	10	2.3	15	3.5

Base: 426 respondents who mentioned answered this item.

n: number of respondents who mentioned this source; %: proportion of respondents who mentioned that source.

### Methods for reaching the nurses at work target population

Among all 650 respondents who completed at least part of the questionnaire, 426 (66%) answered the question on how they had received the nurses at work invitation by mentioning all relevant sources in a closed list and in a comment field. Email (from any source, including graduate mates, 64%) and invitation letters sent by nurses at work (22%) were the most frequently mentioned sources, followed by employers (25%) (Table 3). Facebook and the media were rarely mentioned; however, only one article appeared in the general media and one in the ASI/SBK journal, and our Facebook page was institutional rather individual, therefore less appealing and harder to find.

The questionnaire’s item inquiring whether nurses were still in contact with their graduate mates indicated that out of the 147 nurses who answered that question (only half of the respondents), 60% of the 1988 cohort and 66% of the 1998 cohort reported that they were still in contact with one or more graduate mates. Paradoxically, only 63 respondents (22% of the two test cohorts) provided at least one postal or email address for their graduate mates, indicating a large margin for improving the efficiency of the snowball recruitment process in the main study. Members of the more recent 1998 cohort provided somewhat fewer overall contacts than those from the older 1988 cohort (19% vs. 24%) but more email addresses: 15% (n = 20) vs. 23% (n = 43) respectively indicated at least one postal address, but 11% (n = 12) vs. 8% (n = 14) at least one email address.

### Acceptability of the study

The vast majority of respondents (94%) stated that they would ‘probably’ or ‘maybe’ agree to participate in the main larger nurses at work study (Table 4). There were no notable differences between the two cohorts of 1988 and 1998, or across linguistic regions (data not shown). Nurses working outside nursing or ‘inactive’ were less enthusiastic about future participation, but these last two categories only represented 15 individuals (6% of the respondents from the two test cohorts).

**Table 4:** **Willingness to participate in the larger main nurses at work study, respondents from the two test cohorts of 1988 and 1998 (n = 287)**

		**Probably**		**Maybe**		**Probably not**		**Total**	
		**n**	**%**	**n**	**%**	**n**	**%**	**n**	**%**
	**Total**	**186**	**73.8**	**51**	**20.2**	**15**	**6.0**	**252**	**100.0**
Graduation year	1988	120	75.0	30	18.8	10	6.3	160	100.0
	1998	66	71.7	21	22.8	5	5.4	92	100.0
Current working status	Active in nursing	179	75.5	47	19.8	11	4.6	237	100.0
	Active outside nursing	2	22.2	4	44.4	3	33.3	9	100.0
	Non active	5	83.3	0	0	1	16.7	6	100.0

Base: respondents with complete answers.

Free comments either retrieved from the questionnaire or received through the nurses at work email address were largely positive. Fewer than five respondents stated either that the questions were too limited in scope, or commented on their decision not to communicate other names. Conversely, one respondent forwarded the invitation to all members of her professional specialty association. The surprisingly large number of ‘outsiders’ who answered voluntarily without being part of the target test cohorts is another indication that nurses working in Switzerland have a strong interest in the study’s topic.

### Participants

The questionnaire was available in three languages; 70.9% of test cohort respondents chose French, 24.5% German and 4.6% Italian (Table 5). This distribution reflects the emphasis put in the French- and Italian-speaking regions at the recruitment stage. Overall, 15.5% were male nurses (n = 39). The age distribution reflects the 10-year period between the two cohorts. Median age was expectedly higher for the 1988 than for the 1998 cohort (median 47.4 years, SD 3.7 vs. 39.8 years, SD 5.6).

**Table 5:** **Demographic characteristics of respondents from the two test cohorts of 1988 and 1998 (n = 287)**

		**1988**		**1998**		**Total**	
		**n**	**%**	**n**	**%**	**n**	**%**
Region	French	136	75.6	65	60.7	201	70.0
	German	33	18.3	42	39.3	75	26.1
	Italian	11	6.1	0	.0	11	3.8
	**Total**	**180**	**100.0**	**107**	**100.0**	**287**	**100.0**
Gender	woman	136	84.5	76	84.4	212	84.5
	man	25	15.5	14	15.6	39	15.5
	**Total**	**161**	**100.0**	**90**	**100.0**	**251**	**100.0**
Age (years)	23-39	2	1.3	57	64.8	59	24.4
	40-49	126	81.8	23	26.1	149	61.6
	50+	26	16.9	8	9.1	34	14.0
	**Total**	**154**	**100.0**	**88**	**100.0**	**242**	**100.0**

Region is based on the language chosen by the respondent (no missing value). Totals vary due to missing values on other variables.

Table 6 describes the education curriculum of respondents. Diplomas other than general nursing care represented 17.5% in the 1988 graduation group, and 24.4% for 1998; these diplomas became post-diploma specializations after 1998. Given the poor state of nursing data in Switzerland, it is not possible to assess the representativeness of initial nursing education compared to the whole country for the graduation years under consideration. The majority of nurses (about 72%) have followed some type of continuing education in order to improve their knowledge and skills.

**Table 6:** **Nursing education of the respondents, by graduation year (n = 287)**

	**1988**		**1998**		**Total**	
***Initial diploma type***	**n**	**%**	**n**	**%**	**n**	**%**
General care	142	82.6	78	75.7	220	80
Psychiatric care	18	10.5	14	13.6	32	11.6
Pediatric care (HMP)	12	7.0	11	10.7	23	8.4
**Total**	**172**	**100.0**	**103**	**100.0**	**275**	**100.0**
Continuing education	132	75,4	68	66,0	200	71,9
No continuing education	43	24,6	35	34,0	78	28,1
**Total**	**175**	**100.0**	**103**	**100.0**	**278**	**100.0**

Base: respondents from the two test cohorts.

Overall, and similarly in the two cohorts, most respondents (93.5%, n = 259) were still active in nursing (Table 2). Only 4.3% (n = 12) worked outside nursing, and 2.2% (n = 6) were not currently active in the workforce, irrespective of the cohort or region. These low proportions of nurses non working in nursing probably result from four main factors: the ASI/SBK origin of the seed sample (nurses who quit nursing tend to cancel their membership), the heavy reliance on employer’s advertisement for the study, insufficiencies in our public media communication strategy, and the short duration of the recruitment process.

## Discussion

In absence of a national registry of nurses in Switzerland, we investigated the feasibility of firstly identifying valid denominators, then reaching potential respondents, of a targeted population of nurses 14 or 24 years after graduation, in order to launch the main nurses at work project on career paths and retention.

With the nurses at work feasibility study we were able to approach a substantial number of nurses from two graduation cohorts of nursing schools, allowing us to describe respondent characteristics and willingness for participation in the nurses at work main study. Participation was higher in the French-speaking part of Switzerland, possibly due to the fact that the study originated from this region and might have been considered less attractive to nurses from other linguistic regions.

We were able to determine the size and structure of the target population by comparing two independent sources of graduates’ lists (individual nursing schools and SRC). Although both were found to have advantages and limitations in terms of comprehensiveness, accessibility and ease of file management, we found acceptable discrepancies on aggregate numbers used for denominators that did not impact the computed participation rates. These discrepancies can be reduced or eliminated by investing more clerical efforts on reorganizing some SRC lists that were distributed over two graduation years for administrative reasons. Since legal authorization to access the SRC paper files was only obtained in the last weeks of the study after much negotiation, this could not be done for the pilot study. Getting accurate and valid figures for computing cohort-specific participation rates is a scientific added value. We showed that the centralized SRC source was reliable and timesaving in view of scaling up retrieval efforts for the main nurses at work study from two test cohorts to seven, even though paper files need to be computerized. Only aggregated denominators will be needed for the main study. Individual names at the time of graduation are not useful, since the only usable secondary data source as seed sample turned out to be the ASI/SBK file of members containing reasonably updated contact details.

Acceptability of, and interest for, the study were high among respondents, as demonstrated by the overwhelming willingness of nurses still active in nursing to participate to the main study, by the positive free comments^1^ on the appropriateness of the project, by the large number of respondents who were not part of the two test cohorts (more than twice the latter), and by the warm interest and support demonstrated by employers, in particular in disseminating invitations. This interest must however be moderated by the 26.5% participation rate, modest under the perspective of representativeness, but quite decent when there is no sampling frame such as a Nursing Register. Furthermore, stakeholders and policymakers expressed interest and support (verbally or with funding or in-kind support) for the study, such as Obsan, high-ranking officials from Canton- or Federal-level Public Health or Education Offices, ASI/SBK and SRC, stressing the relevance of the research questions in the context of the nursing shortage. However, our assessment of the acceptability and interest from nurses who left nursing was potentially biased, as they represented only 6% of the respondents.

Recruitment of study participants mostly relied on a snowball procedure, on direct invitations send to ASI/SBK members of the two test cohorts (seed sample), and on invitations disseminated by employers. All these means worked well, but succeeded in reaching mostly nurses working in nursing, as both our seed sample and supporting employers were biased towards such nurses. We failed to reach any significant number of nurses working in other economic sectors or who are not in the workforce anymore. This was further amplified by the weakness of our public communication actions, which were limited in scope and efficiency by the lack of specialized resources in professional communication. Nevertheless, our recruitment process made it possible to reach and recruit nurses from all schools and graduation years of interest, leaving no gap that would be structurally impossible to reach. This is a clear vindication of our retrospective cohort design. We demonstrated that once reached, potential respondents do participate. The intensity and specificity of the recruitment efforts are therefore key, including by motivating the target population for the snowball technique (i.e. to disseminate the invitation widely and to provide additional names of potential participants), by explicitly inviting nurses outside nursing to participate, and by extending the recruitment efforts within the German-speaking part of Switzerland. The strength of obtaining unexpected participation beyond the targeted test cohorts should be built upon. Nursing class leaders of graduation cohorts who keep updated lists of their graduate mates should be especially elicited, as they provided crucial potentially complete lists of nurses when the data collection process was over.

The overall participation rate of 26.5% (up to 40% in French-speaking cantons), based on lists of graduates, can be considered conservative for the reasons developed above, as they were obtained respectively 14 and 24 years after graduation and in the absence of an active national registry of nurses. The participation rate of nurses who were actually invited would be much higher, but cannot be computed since there were multiple simultaneous sources of invitation by design. Higher participation rates were reached in relatively recent studies in countries that maintain national registries of nurses: 61% in the 2008 U.S. National Sample Survey of Registered Nurses (n = 55,151) [26], or 76%-86% in the Danish nurse cohort (N = 23,170 for the first wave) in 1993, 1999 and 2009 [44]. However, the Australia/New-Zealand Nurses and Midwives e-Cohort Study, despite diversified recruitment strategies, only achieved of global response rate 2.3% (N = 334,400) [45]. Participation is also facilitated in studies conducted in health institutions with cross-sectional design, e.g. a response rate of 72.2% for RN4CAST-Switzerland (n = 2,261) [13] or 51.4% (n = 77,681) in the NEXT-Study in 10 EU countries for the first study part (cross-sectional) [46].

Altogether, the main limitations of this study, which will be dealt with for the main study, are the relatively low participation rate, the heterogeneity of the recruitment methods (even if they were each purposely defined and tried in order to optimize our efforts for the main study), and the unequal probability of being represented for the three linguistic regions (all nursing schools for the French- and Italian- speaking regions, but based on a convenience sample for the German-speaking region).

## Conclusion

Results indicate that a larger scale nurses at work study is both acceptable and feasible in the Swiss context, and that the drawback of not having a national registry of nurses can be overcome with a multi-method reach out recruiting strategy. The Federal Office of Public Health is currently working towards creating such a registry and has requested that the experience of the present pilot study be integrated in its definition. The nurses at work study would produce valid scientific information on the career paths of nurses who graduated in Switzerland, to inform policymakers, nursing managers, and scientists in the area of human resources for health. It would contribute to shaping retention interventions based on representative opinions of nurses themselves, whatever their current sector/type of activity, over a large span of time (up to 40 years).

Based on the experience of the nurses at work pilot study, recommendations for the main nurses at work study are:

- to open recruitment to all nurses working in Switzerland (Swiss and other diplomas) as well as to former Swiss nurses, by dropping restrictions which were not well understood; cohorts will be recreated *post hoc*
- to retain and expand the snowball recruitment method, based on the total ASI/SBK members’ file for Switzerland
- with the assistance of a communication specialist, to define a comprehensive and simplified communication strategy with special attention to graduate nurses who are harder to reach (outside nursing or not working)
- to extend the data collection time from nine weeks to five months.

### Endnote

^1^such as « I thank you for investigating the problem and wish with all my heart that one day, working conditions become enjoyable and rewarding again, so that we can say again that nurse is a beautiful profession ; I am, of course, at your disposal. », or « I am very interested in the study results and hope things will be moving forward thanks to it. », or « All the best, I think it is important to try and improve our working conditions, as well as the recognition of our profession. Thank you !!! ».
